# Suppression of MUC1-Overexpressing Tumors by a Novel MUC1/CD3 Bispecific Antibody

**DOI:** 10.3390/antib12030047

**Published:** 2023-07-13

**Authors:** Jun Fang, Shifa Lai, Haoyang Yu, Lan Ma

**Affiliations:** 1 Life Science Division, Graduate School at Shenzhen, Tsinghua University, No. 10, Lishan Road, Nanshan District, Shenzhen 518055, China; 2 BenHealth Biopharmaceutical (Shenzhen) Co., Ltd., No. 10, Gaoxinzhong First Avenue, Nanshan District, Shenzhen 518055, China; shifa.lai@outlook.com (S.L.); guixuanyu@hotmail.com (H.Y.)

**Keywords:** Mucin1, epithelial cancers, oncogenic molecule, immunotherapy, BsAb

## Abstract

Mucin1 (MUC1) is abnormally glycosylated and overexpressed in a variety of epithelial cancers and plays a critical role in tumor progression. MUC1 has received remark attention as an oncogenic molecule and is considered a valuable tumor target for immunotherapy, while many monoclonal antibodies (mAbs) targeting MUC1-positive cancers in clinical studies lack satisfactory results. It would be highly desirable to develop an effective therapy against MUC1-expressing cancers. In this study, we constructed a novel T cell-engaging bispecific antibody (BsAb) targeting MUC1 and CD3 with the Fab-ScFv-IgG format. A high quality of MUC1-CD3 BsAb can be acquired through a standard method. Our study suggested that this BsAb could specifically bind to MUC1- and CD3-positive cells and efficiently enhance T cell activation, cytokine release, and cytotoxicity. Furthermore, our study demonstrated that this BsAb could potently redirect T cells to eliminate MUC1-expressing tumor cells in vitro and significantly suppress MUC1-positive tumor growth in a xenograft mouse model. Thus, T cell-engaging MUC1/CD3 BsAb could be an effective therapeutic approach to combat MUC1-positive tumors and our MUC1/CD3 BsAb could be a promising candidate in clinical applications for the treatment of MUC1-positive cancer patients.

## 1. Introduction

Mucins are large and highly glycosylated proteins and are expressed in many epithelial cells. They play important roles in lubricating and hydrating epithelial cell surfaces and function as a physical barrier against invading pathogens in the epithelium [1]. Mucin1 (also known as MUC1, CD227, EMA, PEM, CA15-3, KL-6, MCD, or MAM6) is the first mucin to be identified and characterized [2]. MUC1 is normally expressed in the apical surface of glandular epithelial cells, including the stomach, kidneys, pancreas, prostate, lungs, and esophagus, and it provides protection to the underlying epithelia [3,4,5]. However, MUC1 is highly overexpressed in a wide range of cancers, including breast cancer, ovarian cancer, pancreatic cancer, colon cancer, and uterine corpus endometrial carcinoma [6]. MUC1 is expressed in more than 90% of breast cancer samples, 25–70% of colon cancer samples, and 10–90% of other forms of cancer tissues. The overexpression of MUC1 is associated with tumor progression and decreased overall survival in patients with various tumors [7,8,9]. In addition, MUC1 expression loses apical–basal polarity, which causes the redistribution of MUC1 over the tumor cell surface [10]. Tumor-associated MUC1 displays the hypo-glycosylation of core glycans, in which the long-branched glycan chains in normal tissues are truncated. The abnormal glycosylation of MUC1 will lead to the formation of tumor-related antigen epitopes (new protein or carbohydrate epitopes) [11,12,13]. Moreover, the aberrant tumor-associated MUC1 can contribute to the change in MUC1 downstream signals through its cytoplasmic domain, which further regulates different aspects of tumor functions (cell growth, proliferation, developmental processes, metastasis, apoptosis, etc.) [14,15,16,17,18]. These features have made tumor-associated MUC1 an ideal target for immunotherapy.

Antibody-based immunotherapy has been developed for cancer treatment over the past few decades and is considered one of the most effective methods to treat solid tumors as well as hematologic malignancies [19]. For the treatment of MUC1-positive tumors, mAbs that target different epitopes of MUC1 have been developed in preclinical and clinical studies. Gatipotuzumab (also known as PankoMab-GEX™ or PMG) is a humanized and glyco-optimized mAb that recognizes tumor-associated MUC1 glycopeptide [20]. It has a high binding affinity and specificity to tumor-associated MUC1, decreased binding to circulating MUC1 from colon and pancreatic cancer patients, and no binding to peripheral blood mononuclear cells (PBMCs) [21]. Gatipotuzumab shows enhanced antibody-dependent cell-mediated cytotoxicity (ADCC) and can induce the apoptosis of MUC1-expressing tumor cells [20]. A Phase I clinical study revealed that gatipotuzumab was safe, well tolerated, and had clinical benefits in MUC1-positive patients with advanced solid tumors, while a Phase II clinical study showed that gatipotuzumab treatment had no obvious benefits over a placebo in tumor patients [22,23]. In another study, the combination therapy of gatipotuzumab with the EGFR-targeting antibody tomuzotuximab showed some anti-tumor activity in heavily pretreated EGFR-positive non-small cell lung cancer (NSCLC) patients and colorectal cancer (CRC) [24]. However, other MUC1-specific mAbs, such as huHMFG1, hPAM4, BrE-3, and CMB-401, always showed limited anti-tumor activity as a monotherapy in clinical studies [25,26,27,28]. To further combat MUC1-positive tumors, one approach that can be tested is bispecific antibodies.

BsAbs can redirect the immune cells to tumor cells by binding antigens on both tumor cells and immune cells, like T cells, natural killer (NK) cells, and macrophages. BsAbs can trigger the cytotoxicity of effector cells toward tumor cells and eliminate tumor cells more effectively [29]. Most BsAbs are designed to target the CD3 complex on T cells since cytotoxic T cells are some of the most potent killer cells of the immune system [30]. For many tumor cell surface antigens, such as EpCAM, CD19, CD20, BCMA, gp100, and GPRC5D, T cell-engaging BsAbs have been successfully developed and have shown significant benefits in clinical studies [31,32,33,34,35,36]. Moreover, an increasing number of BsAbs targeting other tumor antigens (EphA10, Lewis Y, CEA, B7-H4, Her2, etc.) are now under investigation [37,38,39,40,41]. Recently, a clinical study revealed that a MUC1/CD3 bispecific antibody conjugated CIK (cytokine-induced killer) cell therapy could lead to the complete response in primary hepatocellular carcinoma (HCC) patients when combined with PD1 inhibitor treatment [42]. This MUC1/CD3 bispecific antibody was generated via the connection of an anti-CD3 and anti-MUC1 mAb to a PLGA nanoparticle through a chemical reaction, and this BsAb could also elicit anti-tumor activity in lung cancer samples in another study [43].

In this work, we constructed a novel bispecific antibody, which has a monovalent, asymmetric structure and a silenced IgG1 Fc to extend the drug’s half-life. This BsAb can specifically bind to MUC1 and CD3 and promote T cell activation and cytotoxicity. Moreover, our study also showed this BsAb had potent efficacy in inducing T cell-mediated MUC1-positive tumor cell lysis in vitro and inhibiting MUC1-positive tumor growth in the xenograft mouse model. Thus, our findings demonstrated that the MUC1/CD3 BsAb can potentially be developed as a new therapeutic drug in clinical applications for the treatment of MUC1-positive tumor patients.

## 2. Materials and Methods

### 2.1. Cell Lines

HeLa, MCF7, SKOV3, NOZ, and Jurkat were obtained from the Type Culture Collection of the Chinese Academy of Sciences, Shanghai, China, and cultured in DMEM, RPMI-1640, or McCoy’s 5A (Gibco, Waltham, MA, USA) complete medium with 10% fetal bovine serum (FBS, Gibco, Waltham, MA, USA) in a 37 °C humidified incubator containing 5% CO_2_. HEK293F cells were obtained from Kairui Biotech and cultured in the Wayne293™ transfection medium (QuaCell Biotechnology, Zhongshan, China) at 120 rpm in a 37 °C shaker incubator containing humidified air with 5% CO_2_.

### 2.2. Isolation of PBMCs and T Cells

Human PBMCs were obtained from buffy coats of healthy donors by ficoll density gradient centrifugation. Total T cells were purified from the PBMCs using a Pan T cell isolation kit (Miltenyi Biotech, cat. no. 130-096-535, Bergisch Gladbach, Germany) through negative selection according to the manufacturer’s instructions. Briefly, for 10^8^ PBMCs, the cells were resuspended in a 400 µL FACS buffer (1× PBS with 5% FBS) and mixed with a 100 µL Pan T cell Biotin-Antibody Cocktail and incubated at 4 °C for 5 min. Next, a 300 µL FACS buffer and 200 µL Pan T Cell MicroBead Cocktail were added and the cells were incubated at 4 °C for another 10 min. Later, the cell suspension was applied to magnetic separation and the pure T cells were acquired through the depletion of magnetically labeled cells. The purity of the isolated T cells (CD3^+^CD56^−^) was detected via flow cytometry on a BD Accuri C6 plus flow cytometer (>95%). The isolated PBMCs or T cells were cultured in a RPMI-1640 complete medium at 37 °C in a 5% CO_2_ humidified incubator.

### 2.3. Antibody Design and Purification

The MUC1/CD3 BsAb was designed in a Fab-ScFv-IgG format through knob-into-hole technology. The Fab arm (knob) is derived from a humanized anti-MUC1 (hCTMO1) mAb and the ScFv arm (hole) comes from a humanized anti-CD3 (UCHT1) mAb. In the human IgG1 Fc region, several mutations (Leu234Ala/Leu235Ala/Gly237Ala) were introduced to silence Fc effector functions [44]. The expression vector was synthesized and cloned into the pcDNA3.1 vector (Genescript, Nanjing, China) and transfected into HEK293F cells. Briefly, one day prior to transfection, HEK293F cells (2 × 10^6^ cells/mL) were seeded in a Wayne293™ transfection medium. On the day of transfection, the plasmid DNA (MUC1-LC:MUC1-HC-IgG:CD3-ScFv-IgG = 2:3:3) and transfection reagent TA293 (plasmid DNA:TA293 = 1:5) were, respectively, diluted in a KPM medium (Kairui Biotech, Zhuhai, China). Next, the plasmid and TA293 were gently mixed and incubated for 10 min at room temperature. Later, the mixture was added to the culture medium. After 24 h, KE293 and KT-Feed (Kairui Biotech, Zhuhai, China) were added to the culture medium to improve antibody production. After 6–7 days’ culture, the supernatant was collected via centrifugation (4000× *g*, 30 min) and filtered with a 0.22 μm membrane. Antibodies were purified via protein A affinity chromatography (NMab Pro Protein A, Suzhou NanoMicro Technology, Suzhou, China) using ÄKTA avant 25 (Cytiva, Marlborough, MA, USA) followed by size exclusion chromatography (SEC) purification (Chromdex 200 PG, Bestchrom (Shanghai) Biosciences, Shanghai, China). Phosphate-buffered saline (PBS) was used as the mobile phase during SEC purification.

### 2.4. HPLC-SEC and SDS-PAGE Analysis

The purity and integrity of the purified BsAb were detected using HPLC-SEC with Biocore SEC-300 (NanoChrom, Suzhou, China). Purified antibody samples were also applied to SDS-PAGE analysis under non-reducing conditions and reducing conditions. The protein was visualized using Coomassie blue staining. Protein markers (Thermo Scientific, Waltham, MA, USA, Cat# 26610) were loaded as standard controls.

### 2.5. Binding Assay

For cell-based binding analysis, the binding of the MUC1/CD3 BsAb to MUC1 and CD3 antigens was analyzed through flow cytometry using HeLa (MUC1-positive) and Jurkat (CD3-positive) cells. For the negative staining control, NOZ cells were used. 1 × 10^6^ cells per sample were collected via centrifugation at 500× *g* for 5 min and then washed twice with a FACS buffer. The cell pellet was resuspended in 100 µL of a FACS buffer containing the MUC1/CD3 BsAb at various concentrations as indicated and incubated at 4 °C for 30 min followed by washing twice with a FACS buffer. Next, cells were resuspended in a FACS buffer containing a secondary FITC anti-human IgG1 Fc antibody (1:200 dilution, BioLegend, San Diego, CA, USA) and incubated at 4 °C for 30 min, then cells were washed once and resuspended in 200 µL of a FACS buffer and applied to flow cytometry detection on a BD Accuri C6 plus flow cytometer. Finally, for each sample, the median fluorescence intensity (MFI) was counted and analyzed. The binding assay was also performed via ELISA. In brief, the 96-well plate (Thermo Scientific, Waltham, MA, USA) was coated with CD3E&CD3D, MUC1, CEA, NKp46, and IL15RA (Kactus Biosystems, Shanghai, China) at 2 µg/mL and incubated at 4 °C overnight. The next day, the plate was washed three times with a PBST solution (Sigma-Aldrich, Milwaukee, WI, USA), and then 3% BSA-PBST was added and the plate was incubated at 37 °C for 1 h. After incubation, the plate was washed, and the serially diluted MUC1/CD3 BsAb, anti-CD3 mAb, and anti-MUC1 mAb (3-fold dilution, ranging from 0.19 ng/mL to 10 µg/mL) were added and incubated at 37 °C for 1 h. Next, the plate was washed and Goat Anti-Human IgG Fc (HRP) (1:2000 dilution in PBST, Abcam, Waltham, MA, USA) was added and incubated at 37 °C for another 1 h. After incubation, the plate was washed, a TMB solution (Thermo Scientific, Waltham, MA, USA) was added, and it was incubated for 5 min–15 min. Later, 2M H_2_SO_4_ was added to stop the reaction, and the absorbance at 492 nm was detected using a microplate reader (Infinite^®^ F50, Tecan, Mannedorf, Switzerland). 

### 2.6. T Cell Activation Analysis

MUC1-positive HeLa cells were seeded in a 96-well cell culture plate (1 × 10^4^/well) overnight. The next day, the cells were co-cultured with human PBMCs (effector-to-target ratio, E/T = 10:1) in the presence of the serially diluted BsAb (5-fold dilution, ranging from 0.64 ng/mL to 50 µg/mL) and then incubated at 37 °C. After 24 h, the cells were collected and analyzed via flow cytometry using an FITC anti-human CD3 Antibody, a PerCP anti-human CD4 Antibody, an APC anti-human CD8 Antibody, an APC anti-human CD56, a PE anti-human CD25, a PerCP anti-human CD69, and a PE anti-human CD69 (1:40 dilution, BioLegend, San Diego, CA, USA) to test the expression of CD69 and CD25 on CD3^+^CD4^+^ T cells, CD3^+^CD4^+^ T cells, and CD3^−^CD56^+^ NK cells. For CD107a detection, human T cells or PBMCs were co-cultured with HeLa cells (E/T = 10:1), and PE anti-human CD107a Antibodies (1:20 dilution, BioLegend, San Diego, CA, USA) were added to the culture medium. After 1 h of incubation, the GolgiStop solution (Monensin Solution, BioLegend, San Diego, CA, USA) was added and cultured for another 4 h. After incubation, T cells or PBMCs were collected and the expression of CD107a was analyzed via flow cytometry using an FITC anti-human CD3 Antibody, a PerCP anti-human CD4 Antibody, an APC anti-human CD8 Antibody, and an APC anti-human CD56 (1:40 dilution, BioLegend, San Diego, CA, USA). The percentages of CD25, CD69, and CD107a on CD3^+^CD4^+^ T cells, CD3^+^CD8^+^ T cells, and CD3^−^CD56^+^ NK cells were counted and analyzed using GraphPad Prism.

### 2.7. Cytokines Measurement

HeLa cells were seeded in a 96-well cell culture plate (1 × 10^4^/well) overnight. The next day, HeLa cells were co-cultured with human T cells (E/T = 8:1) in the presence of a serially diluted MUC1/CD3 BsAb (5-fold dilution, ranging from 0.64 ng/mL to 50 µg/mL) at 37 °C. After 48 h, the culture supernatant was harvested and the secretions of interferon-γ (IFN-γ), tumor necrosis factor-α (TNF-α), and interleukin-2 (IL-2) were detected through an enzyme-linked immunosorbent assay (ELISA) (Dakewe Biotech, Shenzhen, China) according to the manufacturer’s protocol.

### 2.8. In Vitro Cytotoxicity Assay

Target cells (HeLa, MCF7, SKOV3, and NOZ) were seeded in a 96-well cell culture plate (1 × 10^4^/well) overnight. Human T cells (E/T ratio = 5:1) or PBMCs (E/T ratio = 10:1) were prepared and co-cultured with target cells in the presence of the serially diluted MUC1/CD3 BsAb (5-fold dilution, ranging from 0.64 ng/mL to 50 ug/mL) at 37 °C. After 48 h, cell viability was measured using a Cell Counting Kit-8 reagent (Dojindo Molecular Technologies, Kumamoto, Japan). Briefly, the culture supernatants were removed and the plates were washed twice with a DMEM complete medium. Later, the premixed CCK-8 solution (10-fold diluted in a DMEM complete medium) was added to each well with 100 µL and the plates were incubated at 37 °C for 1–4 h. Finally, the absorbance at 450 nm was measured using a microplate reader (Infinite^®^ F50, Tecan, Switzerland). The cell viability was calculated as (OD450_BsAb+Effector_ − OD450_Medium_)/(OD450_Effector_ − OD450_Medium_) × 100% and the cell viability curve (survival rate) was analyzed using GraphPad Prism. The specific lysis of target cells was also tested via flow cytometry. In brief, HeLa, MCF7, SKOV3, and NOZ cells were dissociated using a trypsin solution (Gibco, Waltham, MA, USA) and washed twice with PBS. After washing, the cells were resuspended with 1 mL of PBS and Cell Proliferation Dye eFluor™ 670 (eBioscience, San Diego, CA, USA) was added at a final concentration of 5 µM, and then the cells were incubated at 37 °C for 5 min. Later, the cells were washed twice with a cold DMEM complete medium and seeded in a 24-well cell culture plate (1 × 10^5^/well). Human T cells (E/T ratio = 5:1) were added and co-cultured with the serially diluted MUC1/CD3 BsAb (5-fold dilution, ranging from 0.64 ng/mL to 50 µg/mL) at 37 °C. After 24 h, the cells were collected and stained with 7-AAD (1:300 dilution in a FACS buffer, BioLegend, San Diego, CA, USA) and then applied to flow cytometry detection. 

### 2.9. In Vivo Efficacy Study

Female B-NDG (NOD.CB17-Prkdc^scid^Il2rg^tm1^/Bcgen) mice (6 weeks) were purchased from Biocytogen Pharmaceuticals and fed in accordance with guidelines from the Institutional Animal Care and Use Committee (IACUC) of the Shenzhen Center for Disease Control and Prevention. Each B-NDG mouse was subcutaneously (s.c.) injected with 2.5 × 10^6^ HeLa cells (0.2 mL PBS) in the right flank. About 9 days later, the tumor volume reached 60~80 mm^3^ and the mice were randomized into three groups: a tumor-only group, PBS (vehicle)-treated group, and BsAb-treated group. For the last two groups, each mouse was given 1 × 10^7^ T cells via intraperitoneal (i.p.) injection. Next, the mice were intraperitoneally administrated with 200 µL of PBS or 200 µL of BsAb’s (250 µg/mL), respectively, twice a week for a total of 7 doses. The mouse body weight and tumor size were recorded weekly. The tumor volume was calculated as 1/2 × (length × width × width). Then, 45 days later, mice were sacrificed and the tumor weight was measured using electronic analytical balance.

### 2.10. Statistical Analysis

Data were reported as the mean ± SEM. A two-tailed Student’s *t*-test was used to compare the differences between samples as indicated in the figures. GraphPad Prism version 6.0 (GraphPad Software Inc., San Diego, CA, USA) was used to calculated the data. A value of *p* < 0.05 was considered to be statistically significant.

## 3. Results

### 3.1. Design and Generation of MUC1/CD3 BsAb

**Figure 1:**
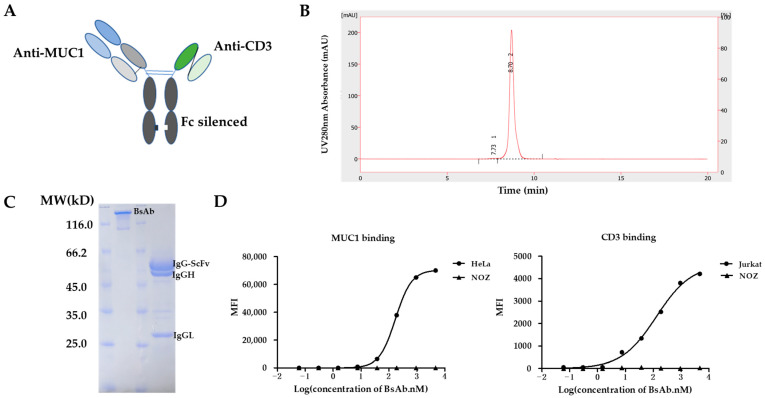
Generation of MUC1/CD3 BsAb. (**A**) Schematic design of MUC1/CD3 BsAb. (**B**) SEC-HPLC analysis of the purified MUC1/CD3 BsAb. The percentage of the area of peak 1 and peak 2 is 0.7% and 99.3% respectively, which represents the high purity of the purified BsAb. (**C**) SDS/PAGE and Coomassie blue staining results of the purified MUC1/CD3 BsAb under non-reducing and reducing conditions. (**D**) Cell binding analysis of MUC1/CD3 BsAb to MUC1 and CD3-positive cells by flow cytometry, HeLa (MUC1-positive cells), Jurkat (CD3-positive cells), NOZ (CD3 and MUC1-negative cells).

The MUC1/CD3 BsAb was designed in a Fab-ScFv-IgG format (Figure 1A). The anti-CD3 ScFv (UCHT1; hole) and the anti-MUC1 Fab (hCTMO1; knob) form a heterodimer through the knob-into-hole strategy, in which the CH3 knob harbors the S354C/T366W mutations and the CH3 hole contains the Y349C/T366S/L368A/Y407V mutations. The human IgG1 Fc part contributes to an extended half-life and is modified with Leu234Ala/Leu235Ala/Gly237Ala mutations, which abrogates its binding to Fc gamma receptors (FcγR) and complement component (C1q) and prevents the antibody-dependent cell-mediated cytotoxicity (ADCC), antibody-dependent cellular phagocytosis (ADCP), and complement-dependent cytotoxicity (CDC) activity, with T cells being the only immune effector cells engaged by the MUC1/CD3 BsAb. MUC1/CD3-BsAb-expressing vectors were transfected in HEK293F cells and the BsAb was first isolated via Protein A affinity chromatography and then purified via size exclusion chromatography. SEC-HPLC analysis showed that the purified MUC1/CD3 BsAb had a high purity and the aggregation level of the MUC1/CD3 BsAb was less than 1% (Figure 1B). The purified BsAb was further applied to SDS-PAGE analysis under either non-reducing or reducing conditions, followed by Coomassie blue staining. The staining results revealed the bands of the expected size of the BsAb on both non-reducing gels (full BsAb: ~127 kD) and reducing gels (IgGL: ~25 kD, IgGH: ~50 kD, IgG-ScFv: ~52 kD), which suggested the purified BsAb had a high integrity and purity (Figure 1C). These results demonstrated that a high-quality MUC1/CD3 BsAb can be efficiently obtained using a standard method. Later, we analyzed the binding specificity of the MUC1/CD3 BsAb via flow cytometry and ELISA assay. The MUC1/CD3 BsAb can specifically bind to MUC1-positive HeLa cells and CD3-positive Jurkat cells in a BsAb dose-dependent manner, while the MUC1/CD3 BsAb cannot bind to the MUC1- and CD3-negative NOZ cells (Figure 1D). Moreover, the MUC1/CD3 BsAb can specifically bind to the CD3E&CD3D antigen and MUC1 antigen as its parental anti-CD3 and anti-MUC1 mAb and cannot bind to unrelated antigens (CEA, NKp46, and IL15RA) in the ELISA (Figure S1) assay. Therefore, this MUC1/CD3 BsAb can maintain the binding specificity of the parental mAb.

### 3.2. MUC1/CD3 BsAb Effectively Induces T Cell-Mediated Lysis of MUC1-Positive Tumor Cells In Vitro

**Figure 2:**
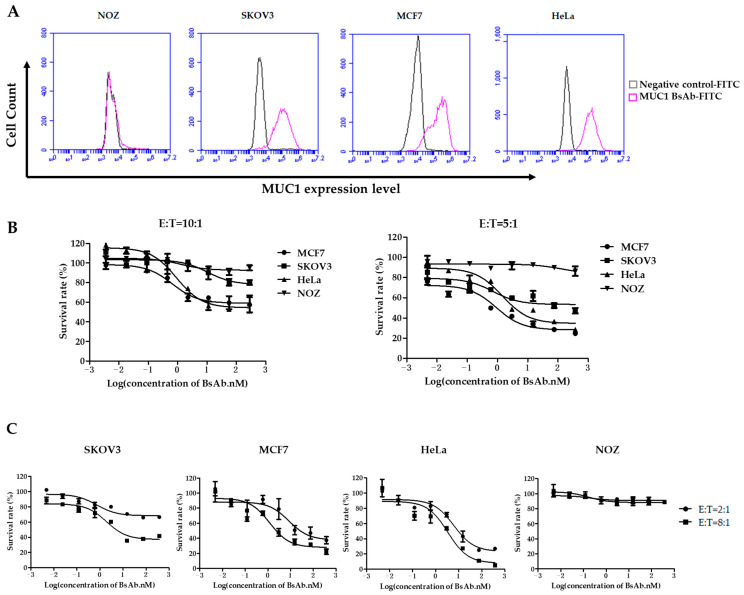
MUC1/CD3 BsAb can induce potent T cell-mediated MUC1-expressing cell lysis. (**A**) Tumor cell lines: HeLa, MCF7, SKOV3, and NOZ were first stained by MUC1/CD3 BsAb and followed by a secondary FITC anti-human IgG Fc antibody. MUC1 expression was detected by flow cytometry. (**B**) Detection of tumor cell lysis after 48 h of incubation with human PBMCs (E/T 10:1) or T cells (E/T 5:1) with serially diluted antibodies. (**C**) Detection of T cell-mediated cytotoxicity in the presence of serially diluted antibodies with different E/T ratios (2:1 or 8:1) in human T cell and tumor cell co-culture cytotoxic assay.

To evaluate whether MUC1/CD3 BsAb can mediate T cell-directed MUC1-positive tumor cell lysis in vitro, the cell cytotoxic assay was performed for MUC1-positive or negative tumor cells. First, a panel of tumor cell lines was analyzed by flow cytometry for MUC1 surface expression. SKOV3, MCF7, and HeLa cells have high MUC1 expression, while NOZ cells have little MUC1 expression (Figure 2A). Next, the purified human PBMCs (E/T = 10:1) or T cells (E/T = 5:1) were co-cultured with tumor cells in the presence of serially diluted BsAb. The MUC1/CD3 BsAb can efficiently induce T cell-mediated lysis of the MUC1-positive cells (i.e., SKOV3, MCF7, and HeLa) and this T cell-mediated cytotoxicity was in a BsAb dose-dependent manner and the BsAb cannot induce T cell-mediated cytotoxicity against MUC1-negative NOZ cells (Figure 2B). This result suggested that the killing capacity induced by MUC1/CD3 BsAb was in a MUC1-specific manner. Moreover, increasing the effector-to-target cell ratio from 2:1 to 8:1 in human T cells and tumor cells co-culture assay can further improve the T cell-mediated cytotoxicity toward MUC1-positive tumor cells (Figure 2C). Furthermore, cell cytotoxic assay was also performed by flow cytometry and the MUC1/CD3 BsAb can induce T cell-mediated killing of MUC1-positive tumor cells in a similar pattern (Figure S2). These results demonstrated that the MUC1/CD3 BsAb can efficiently induce T cell-mediated lysis of MUC1-positive tumor cells in vitro.

### 3.3. MUC1/CD3 BsAb Potently Activates T Cell In Vitro

**Figure 3:**
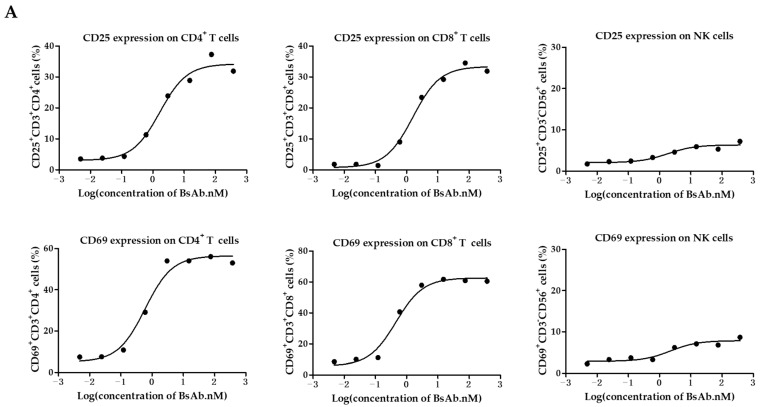

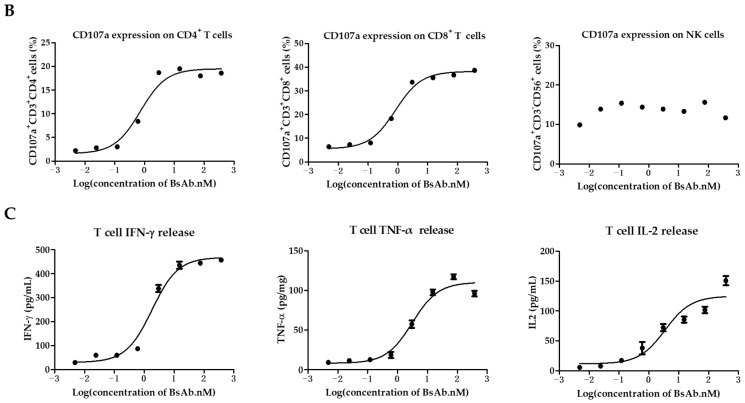
MUC1/CD3 BsAb can induce robust T cell activation. (**A**) Detection of BsAb-induced T cell and NK cell activation (CD69 and CD25). HeLa cells were co-cultured with human PBMCs with an effector-to-target ratio of 10:1 for 24 h with serially diluted antibodies. After incubation, PBMCs were collected, stained, and analyzed for CD69 and CD25 expression via flow cytometry. (**B**) Detection of BsAb-induced CD107a expression on CD3^+^CD4^+^ T cells, CD3^+^CD8^+^ T cells, and NK cells. HeLa cells were co-cultured with human T cells or PBMCs with an effector-to-target ratio of 10:1 with various concentrations of BsAb as indicated, then a CD107a antibody was added to the culture medium. After another 4 h, cells were collected and analyzed for CD107a expression via flow cytometry. For detailed gating strategy of CD25, CD69, and CD107a expression, please refer to the supplementary materials. (**C**) Measurement of cytokine produced in the co-culture supernatant via ELISA. HeLa cells were co-cultured with human T cells with an effector-to-target ratio of 8:1 in the presence of serially diluted BsAb. After 48 h of incubation, cell-free supernatant was obtained and the secretion of IFN-γ, TNF-α, and IL-2 was detected via ELISA. The data shown here are representative of three individual experiments.

We further examined the function of MUC1/CD3 BsAb by evaluating its ability to activate T cells. First, the freshly isolated PBMCs were co-cultured with HeLa cells in the presence of serially diluted BsAb for 24 h, then the PBMCs were collected and analyzed for T cell and NK cell activation by flow cytometry. The results showed that the MUC1/CD3 BsAb can upregulate CD69 and CD25 expression on CD3^+^CD4^+^ T cells and CD3^+^CD8^+^ T cells in a BsAb dose-dependent manner in the co-culture assay, while the MUC1/CD3 BsAb had little effects in inducing CD69 and CD25 expression on CD3^−^CD56^+^ NK cells (Figure 3A). Cell surface CD107a expression has been widely used to measure T cell activation and cytotoxic function. Here we also found that the MUC1/CD3 BsAb can efficiently induce CD107a expression on CD3^+^CD4^+^ T cells and CD3^+^CD8^+^ T cells in an antibody dose-dependent manner, while the MUC1/CD3 BsAb cannot induce CD107a expression on CD3^−^CD56^+^ NK cells (Figure 3B). To further detect MUC1/CD3 BsAb induced T cell activation upon target cell lysis, cytokines produced in the supernatant of T cells and HeLa cells co-culture assay were examined via an ELISA assay. We found the MUC1/CD3 BsAb can significantly enhance T cell cytokine (IFN-γ, TNF-α, and IL-2) production, and the cytokine production was in a BsAb dose-dependent manner (Figure 3C). These results suggested that our MUC1/CD3 BsAb can robustly activate T cells and enhance T cell cytotoxicity in vitro.

### 3.4. MUC1/CD3 BsAb Efficiently Inhibits MUC1-Positive Tumor Cell Growth In Vivo

**Figure 4:**
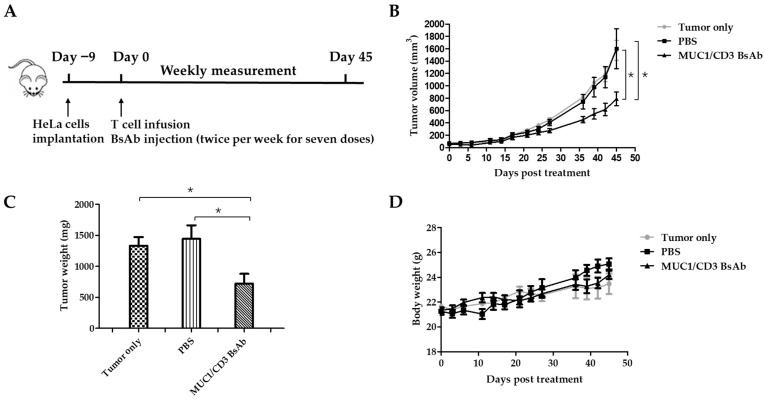
MUC1/CD3 BsAb can efficiently suppress MUC1-expressing tumors in B-NDG mice. (**A**) Schematic schedule of the in vivo study. Each B-NDG mouse was engrafted with 2.5 × 10^6^ HeLa cells in the right flank via subcutaneous injection on day −9. On day 0, the mice were randomized into three groups as indicated. For BsAb- and PBS (vehicle)-treated groups, each mouse was intraperitoneally injected with 1 × 10^7^ T cells. One hour later, the mouse was given 200 µL of a BsAb (250 µg/mL) solution or PBS via intraperitoneal injection, respectively. The PBS or antibody treatment was given twice per week for a total of 7 doses. Mice body weights and tumor sizes were measured twice per week. (**B**) Time course of tumor growth of different groups. (**C**) Tumor weight comparison between different groups. (**D**) Time course of body weight of different groups. Experimental data are presented as mean ± SEM (*p* < 0.05 (*)).

Finally, the in vivo efficacy of the MUC1/CD3 BsAb was examined in a xenograft mouse model. B-NDG mice were engrafted with HeLa cells in the right flank via subcutaneous injection. About 9 days later, the tumor-bearing mice were randomly assigned into three groups: the tumor-only group, PBS (vehicle)-treated group, and BsAb-treated group. For the PBS- and BsAb-treated groups, each mouse was intraperitoneally injected with 1 × 10^7^ T cells, then the BsAb-treated group was intraperitoneally injected with a BsAb solution and the PBS-treated group received an intraperitoneal injection of PBS. The mice were treated twice weekly with an antibody solution or PBS for a total of 7 doses. To evaluate anti-tumor efficacy, the tumor volume was measured weekly. About forty-five days later, the mice were sacrificed and the tumor tissues were collected and weighed (Figure 4A). Consistent with the in vitro cytotoxicity assay, MUC1/CD3 BsAb treatment can significantly inhibit MUC1-positive tumor cell growth in the xenograft mouse model (Figure 4B,C). Furthermore, there was no significant difference in body weight between antibody- and PBS-treated mice, which suggested the BsAb had no apparent adverse effects (Figure 4D). These results demonstrated that the MUC1/CD3 BsAb can efficiently suppress MUC1-positive tumors’ growth in vivo.

## 4. Discussion

Targeting tumor-associated mucins for immunotherapy has gained increasing attention due to their abnormal expression and critical roles in tumor progression. MUC1 is one of the most attractive tumor antigens for the immunotherapy of various tumors [11]. Many mAbs have been developed to target different MUC1 epitopes for the treatment of MUC1-positive tumors, while most of these antibodies did not show clinical benefits as monotherapy in MUC1-positive tumor patients [45]. In recent years, the use of BsAbs has been proven as a powerful approach for the treatment of cancer and other diseases. Different formats of BsAbs have been constructed and studied in preclinical and clinical studies [46]. Blinatumomab, a CD19/CD3 BiTE, has been successfully applied to treat acute lymphoblastic leukemia and shows significant clinical benefits, though it needs continuous intravenous infusion due to its short half-life [47]. T cell-engaging BsAbs still face many challenges in the application for tumor treatment, such as cytokine release syndromes, neurotoxicity, and off-target toxicities [48,49]. Moreover, the developability and druggability of BsAbs are usually more challenging than conventional mAbs [50]. As a promising approach for cancer treatment, many strategies have been adopted to make the BsAb more applicable.

In this study, we constructed a novel T cell-engaging BsAb targeting MUC1 and the CD3 antigen, which can bind T cells and tumor cells simultaneously and redirect T cells to kill tumor cells. This BsAb was designed in an asymmetric Fab-ScFv-IgG format with a Fab arm binding to MUC1 on tumor cells and a ScFv arm recognizing CD3 on T cells. Knob-into-hole technology was adopted to avoid the formation of homodimers. Several mutations were introduced in the IgG Fc region, which retained the binding ability to the FcRn receptor (neonatal Fc receptor) for a long half-life and lost binding ability to FcγR and C1q to avoid potential ADCC, ADCP, and CDC effects [44]. The MUC1/CD3 BsAb was expressed in HEK293F cells and purified via protein A affinity chromatography and size exclusion chromatography. The purified BsAb had a high integrity and purity as tested using SEC-HPLC and Coomassie blue staining. Our results demonstrated that this BsAb can specifically bind to CD3- and MUC1-positive cells as detected via flow cytometry and this BsAb showed specific binding to the CD3E&CD3D antigen and MUC1 antigen and no binding to unrelated antigens as tested via an ELISA assay. The MUC1/CD3 BsAb exhibited potent efficiency in inducing T cell activation and T cell cytotoxicity by upregulating CD69, CD25, and CD107a expression in both CD4^+^ T cells and CD8^+^ T cells, while it had little effects on NK cells in the co-culture assay. This BsAb can robustly induce cytokines’ (IFN-γ, TNF-α, and IL-2) secretion in T cells and MUC1-expressing tumor cells’ co-culture assay. In addition, our study revealed that the BsAb could efficiently and specifically induce T cell-mediated MUC1-positive tumor cell (HeLa, MCF7, SKOV3) lysis in vitro in a BsAb dose-dependent manner. Furthermore, our study showed the MUC1-CD3 BsAb can potently suppress MUC1-positive tumor growth in a xenograft mouse model.

We have proved that our BsAb has potent efficiency in inducing the T cell-mediated killing of several MUC1-expressing tumors (cervical cancer, breast cancer, and ovarian carcinoma) and this MUC1/CD3 BsAb could potentially be developed as a therapeutic antibody drug for the treatment of MUC1-expressing tumors, such as breast cancer, ovarian cancer, pancreatic cancer, colon cancer, cervical cancer, and uterine corpus endometrial carcinoma. Thus, our findings provide meaningful evidence that T cell-engaging BsAb can be an effective therapeutic approach for MUC1-positive tumors. Moreover, the immune checkpoint inhibitors (anti-PD1, anti-PDL1) have been successfully applied in clinical applications, and the combination of immune checkpoint inhibitors with the BsAb has been studied in many preclinical and clinical studies [51]. A recent study suggested that a MUC1/CD3 BsAb (PLGA nanoparticle connected with an anti-MUC1 mAb and anti-CD3 mAb) conjugated CIK cell therapy showed encouraging clinical results in hepatocellular carcinoma patients when combined with anti-PD1 treatment [42]. Therefore, in future work, the anti-tumor efficacy of MUC1/CD3 BsAb in combination with the immune checkpoint inhibitors may be worth studying.

## 5. Conclusions

Our findings suggested that the MUC1/CD3 BsAb in a Fab-ScFv-IgG format can be produced with a high integrity and purity. It can maintain the specific binding to MUC1 and CD3 as the parental anti-CD3 and anti-MUC1 mAb. This MUC1/CD3 BsAb can potently promote CD4^+^ T and CD8^+^ T cell activation and cytotoxicity by upregulating CD69, CD25, and CD107a expression and inducing T cell cytokine secretion in the co-culture assay. Our MUC1/CD3 BsAb can efficiently redirect T cells to kill MUC1-positive tumor cells in vitro and in vivo. Thus, the T cell-engaging BsAb could be an effective therapeutic approach for MUC1-positive tumors and our MUC1/CD3 BsAb could be developed as a promising therapeutic drug for MUC1-positive tumors’ treatment in clinical applications.

## Data Availability

The dataset supporting the conclusions of this article is included within the article.

## Supplementary Materials

The following supporting information can be downloaded at: https://www.mdpi.com/article/10.3390/antib12030047/s1, Figure S1: MUC1/CD3 BsAb specifically binds to CD3E&CD3D antigen and MUC1 antigen. Detection the binding of MUC1/CD3 BsAb, anti-MUC1 mAb, and anti-CD3 mAb to related antigens (CD3E&CD3D antigen and MUC1 antigen) and unrelated antigens (CEA, NKp46, and IL15RA) by ELISA. MUC1/CD3 BsAb can specifically bind to CD3E&CD3D antigen and MUC1 antigen as its parental anti-CD3 mAb and anti-MUC1 mAb, while MUC1 cannot bind to unrelated antigens (CEA, NKp46, and IL15RA). Figure S2: MUC1/CD3 BsAb induces T cell-mediated specific lysis of MUC1-expressing tumor cells. The cytotoxic assay was performed by flow cytometry. Tumor cells (MUC1-positive cells: HeLa, MCF7, and SKOV3; MUC1-negative cells: NOZ) were labeled with Cell Proliferation Dye eFluor™ 670 and incubated with human T cells (E/T ratio = 5:1) in the presence of serially diluted BsAb at 37 °C for 24 h. After incubation, cells were collected and stained with 7-AAD solution. eFluor™ 670 and 7-AAD double-positive cells represent dead tumor cells. eFluor™ 670-positive and 7-AAD-negative cells represent live tumor cells. The gating strategy and raw data are listed below.
