# Supplementary material for: Suppression of MUC1-Overexpressing Tumors by a Novel MUC1/CD3 Bispecific Antibody
Source: Antibodies (Basel). 2023 Jul 13;12(3):47. doi: 10.3390/antib12030047 (PMC10366937; doi:10.3390/antib12030047)
Supplement: Supplementary file 1 [file antibodies-12-00047-s001.zip › antibodies-2454615-supplementary.pdf]

Supplementary data

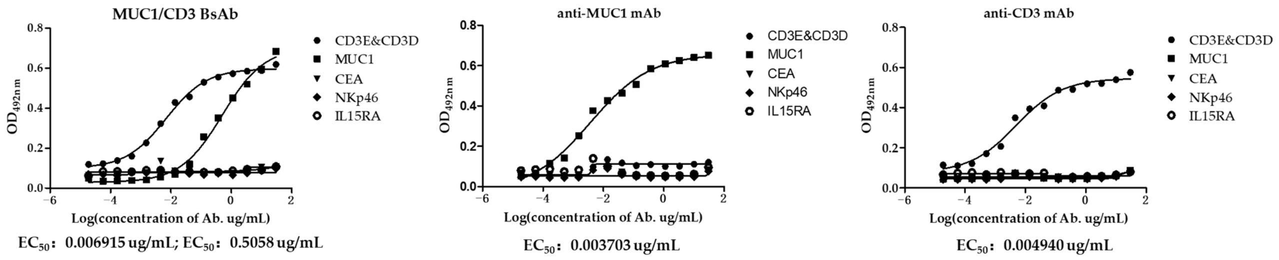

Figure S1. MUC1/CD3 BsAb specifically binds to CD3E&CD3D antigen and MUC1 antigen. Detection the binding of MUC1/CD3 BsAb, anti-MUC1 mAb, and anti-CD3 mAb to related antigens (CD3E&CD3D antigen and MUC1 antigen) and unrelated antigens (CEA, NKp46, and IL15RA) by ELISA. MUC1/CD3 BsAb can specifically bind to CD3E&CD3D antigen and MUC1 antigen as its parental anti-CD3 mAb and anti-MUC1 mAb, while MUC1 cannot bind to unrelated antigens (CEA, NKp46, and IL15RA).

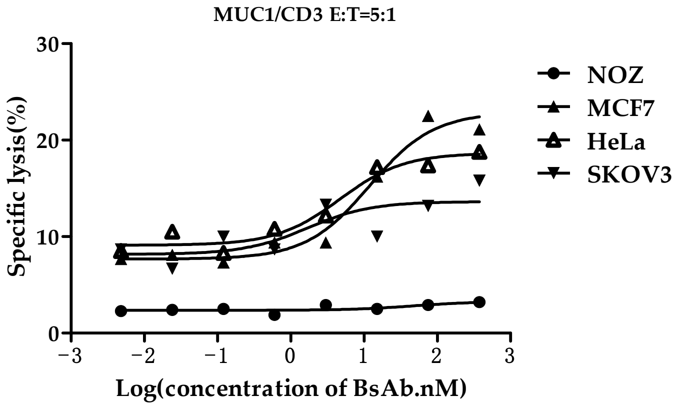

Figure S2. MUC1/CD3 BsAb induces T cell-mediated specific lysis of MUC1-expressing tumor cells. The cytotoxic assay was performed by flow cytometry. Tumor cells (MUC1-positive cells: HeLa, MCF7, and SKOV3; MUC1-negative cells: NOZ) were labeled with Cell Proliferation Dye eFluor™ 670 and incubated with human T cells (E/T ratio=5:1) in the presence of serially diluted BsAb at 37°C for 24h. After incubation, cells were collected and stained with 7-AAD solution. eFluor™ 670 and 7-AAD double-positive cells represent dead tumor cells. eFluor™ 670-positive and 7-AAD-negative cells represent live tumor cells. The gating strategy and raw data are listed below.

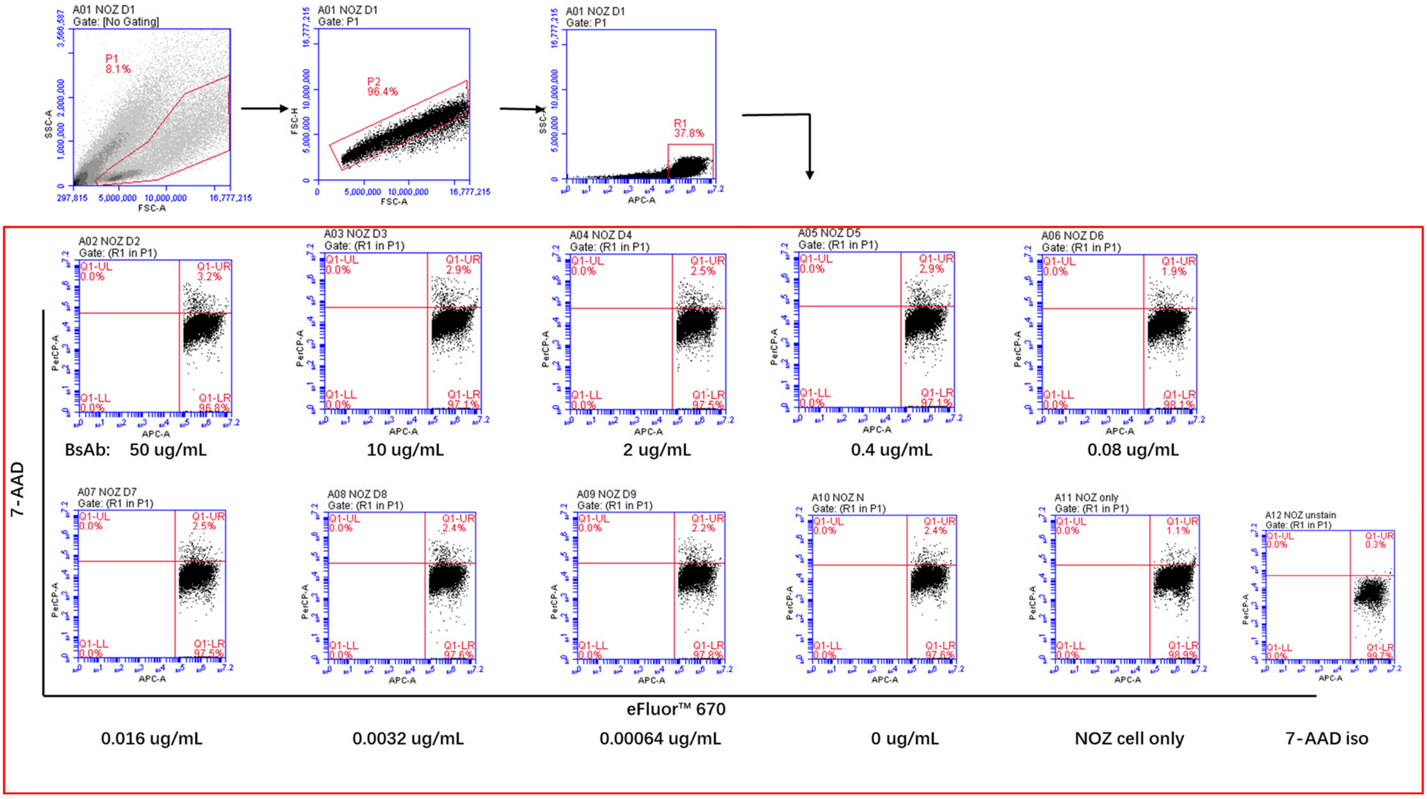

Cytotoxic assay for NOZ cells by flow cytometry

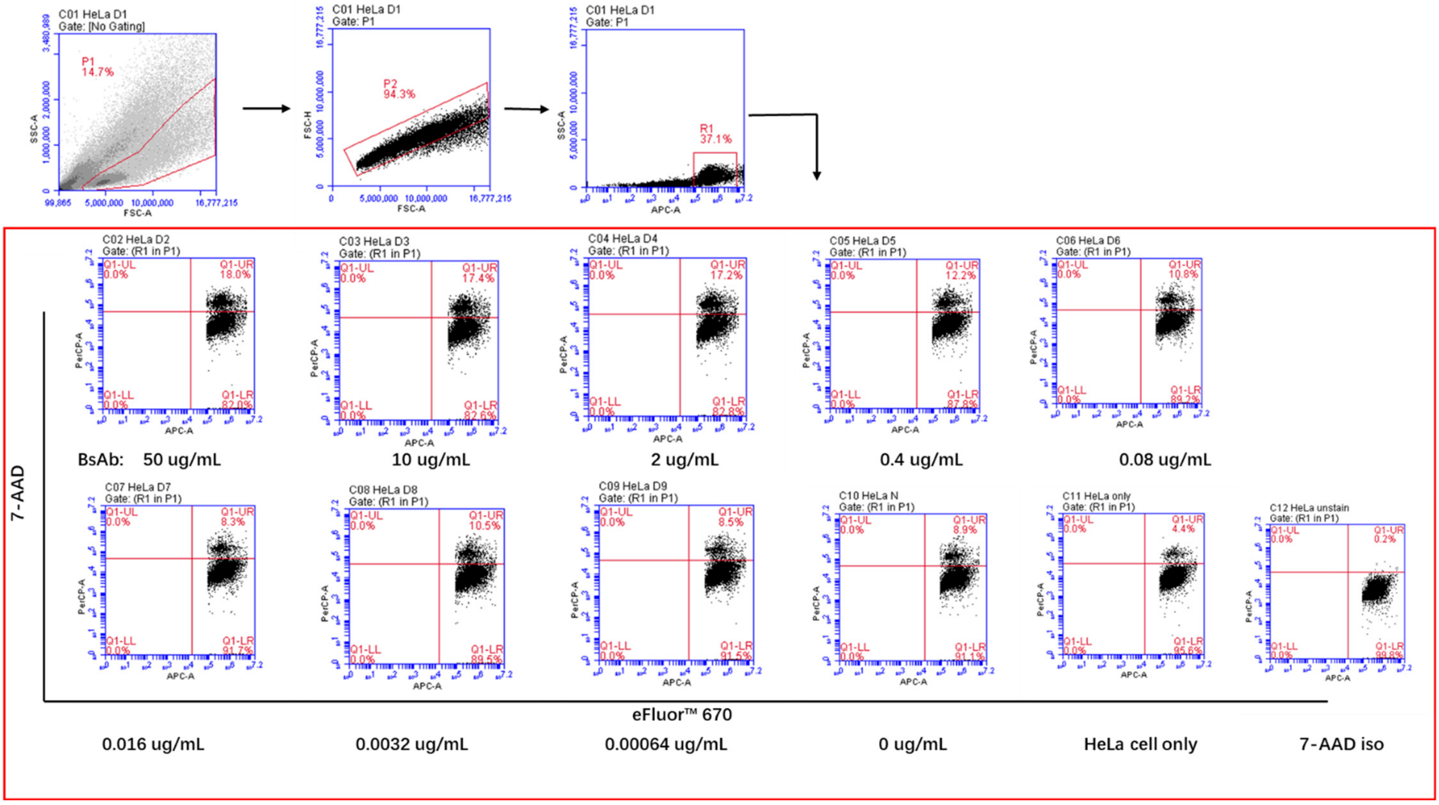

Cytotoxic assay for HeLa cells by flow cytometry

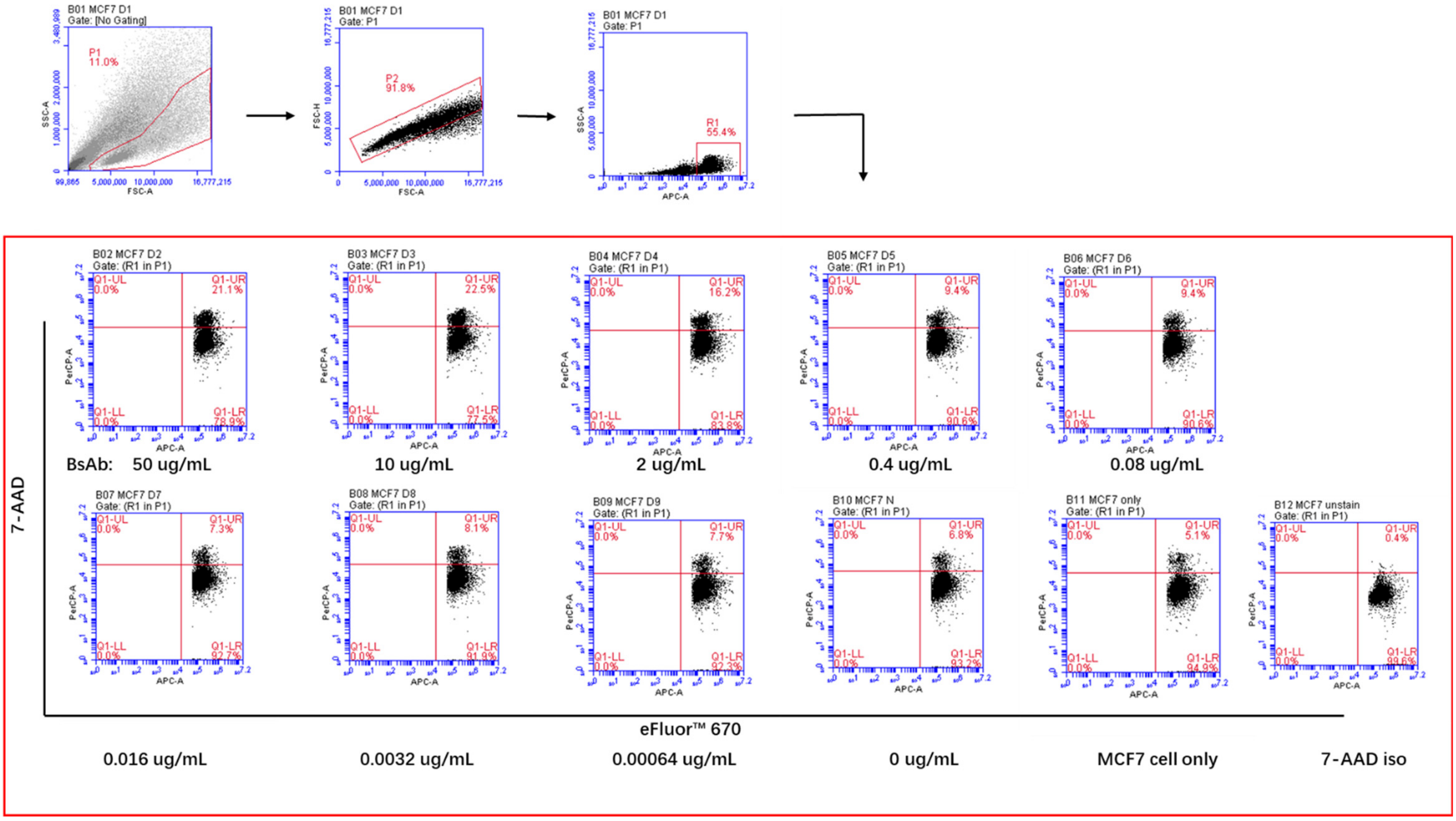

Cytotoxic assay for MCF7 cells by flow cytometry

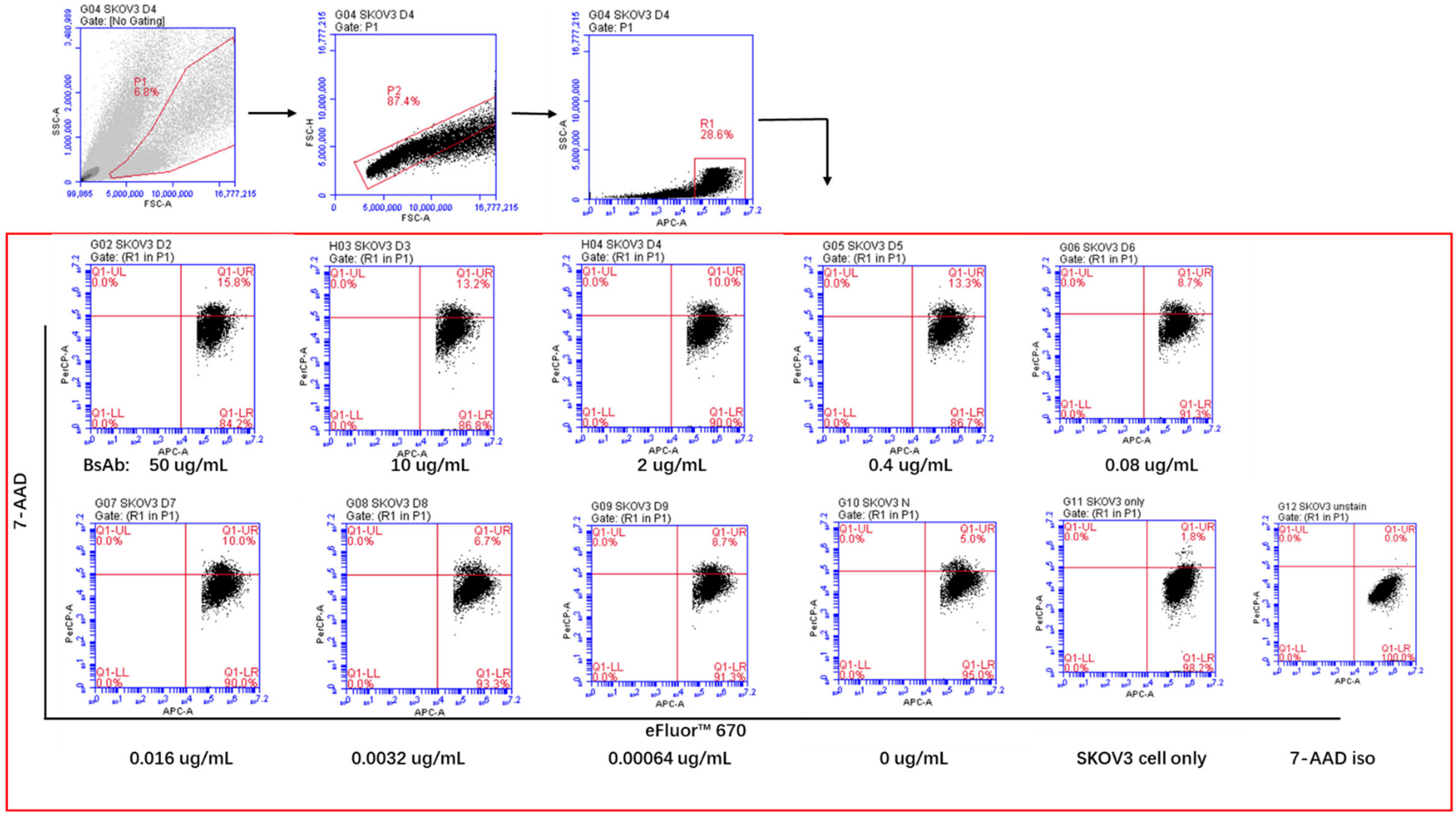

Cytotoxic assay for SKOV3 by flow cytometry

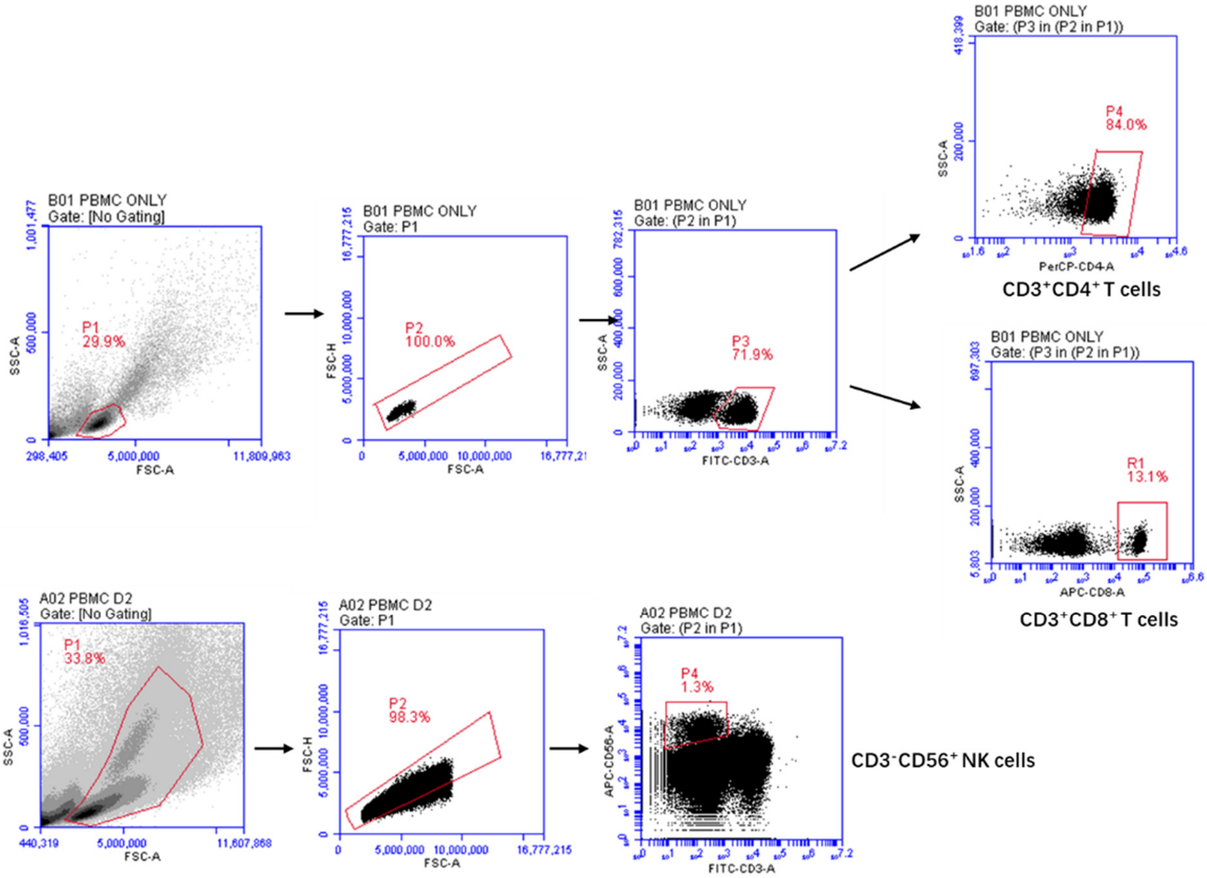

Gating strategy for CD3⁺CD4⁺ T cells, CD3⁺CD8⁺ T cells, and CD3⁻CD56⁺ NK cells for the detection of CD25, CD69, and CD107a expression.

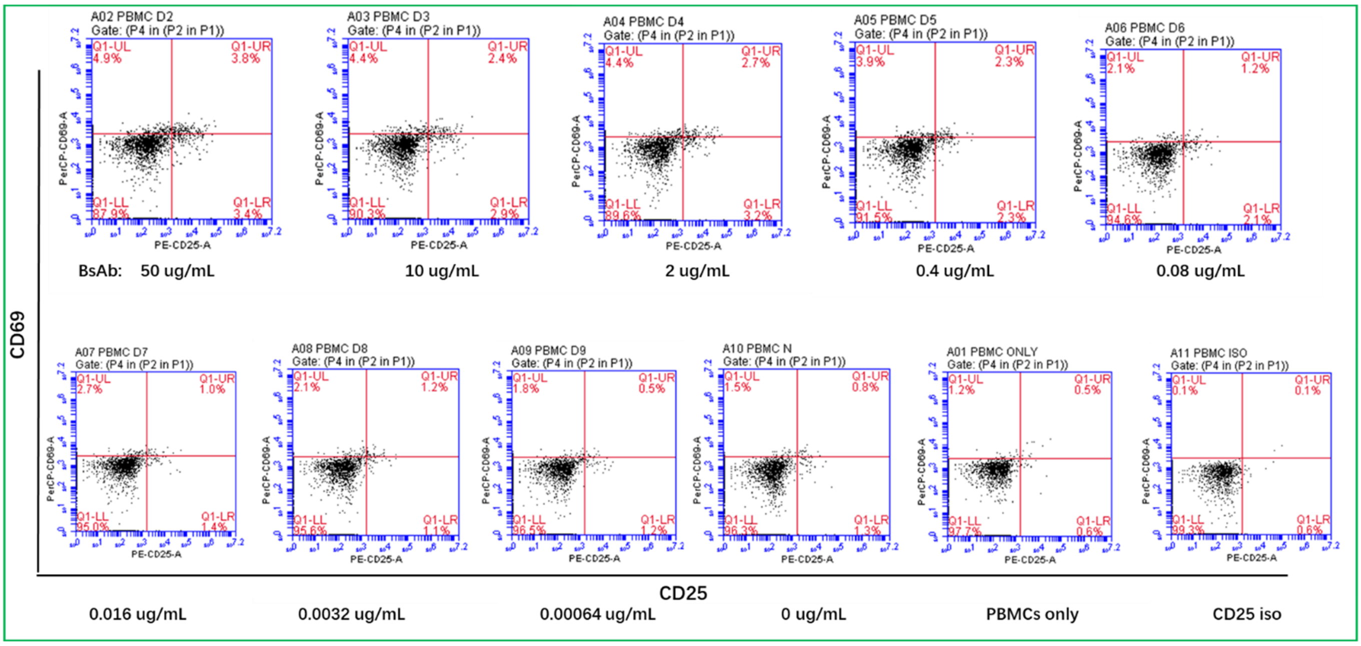

CD25 and CD69 expression on CD3⁻CD56⁺ NK cells

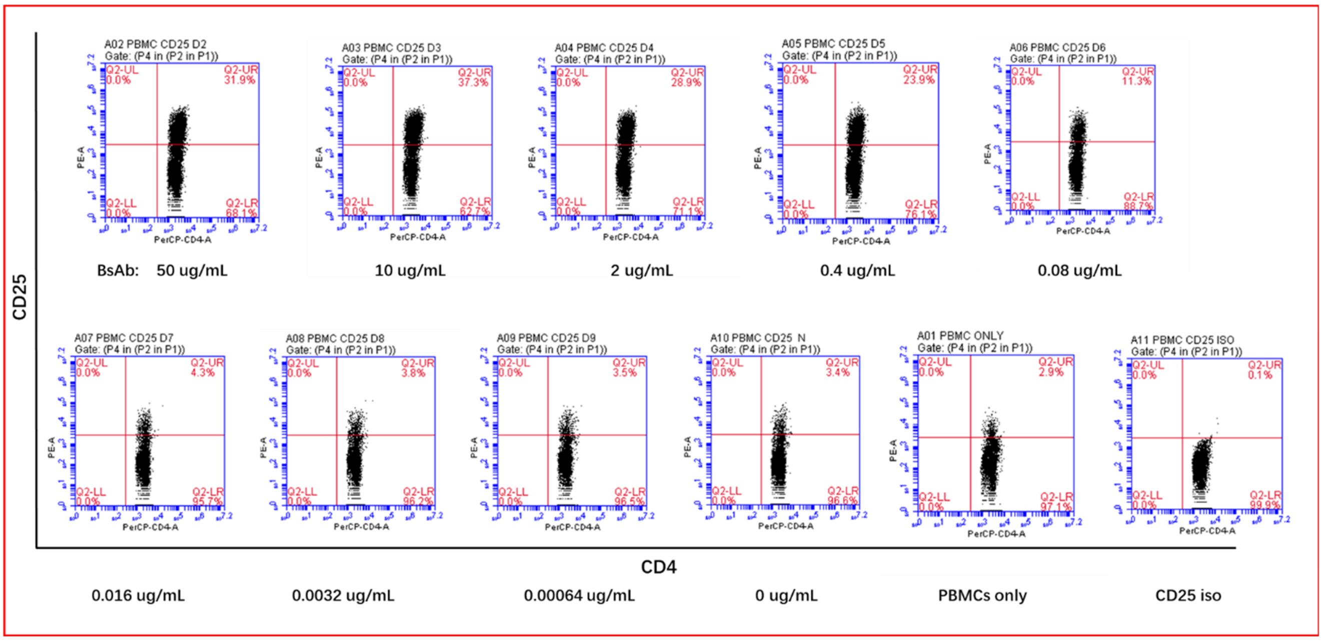

CD25 expression on CD4+ T cells

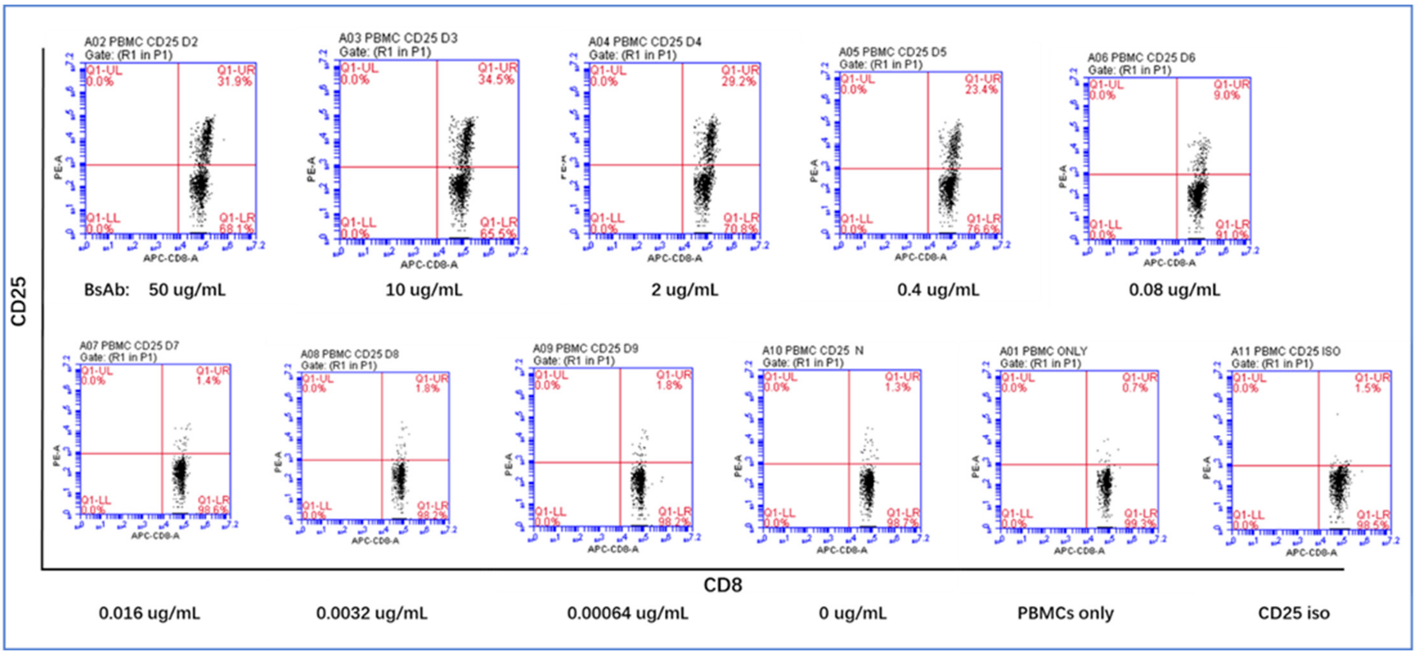

CD25 expression on CD8+ T cells

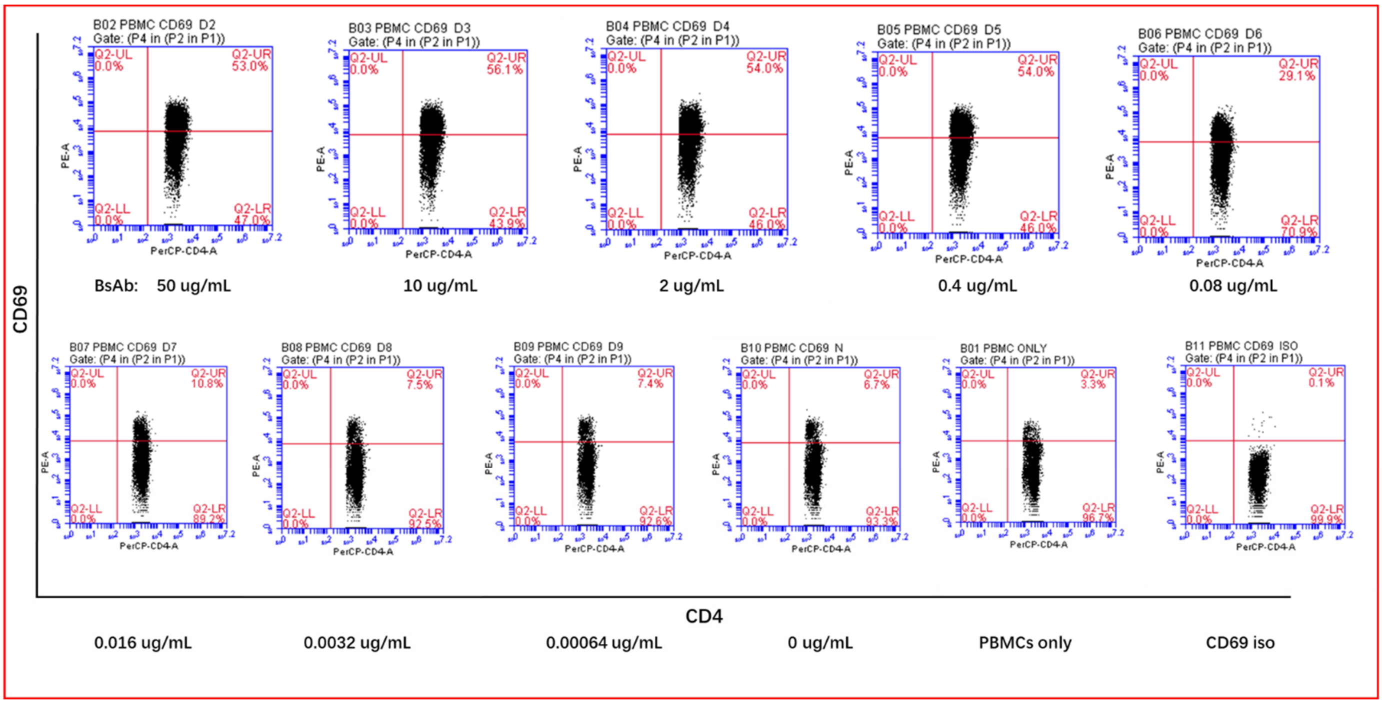

CD69 expression on CD4+ T cells

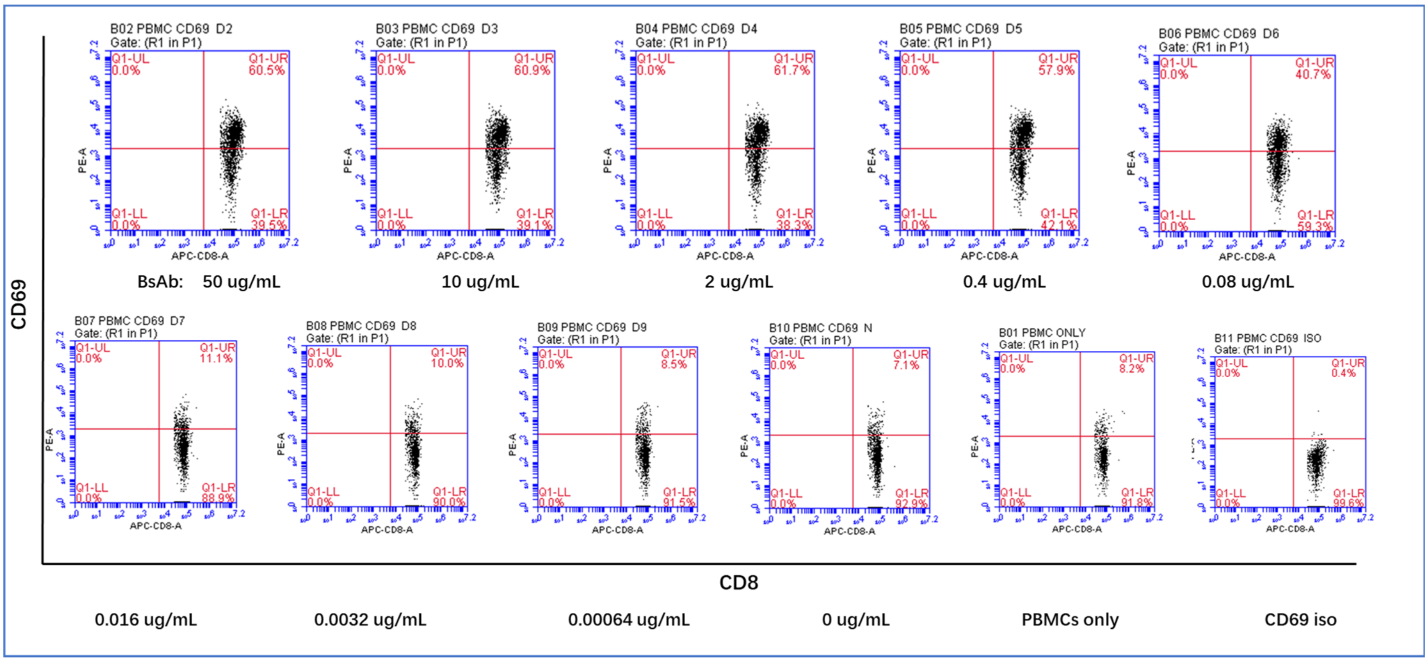

CD69 expression on CD8+ T cells

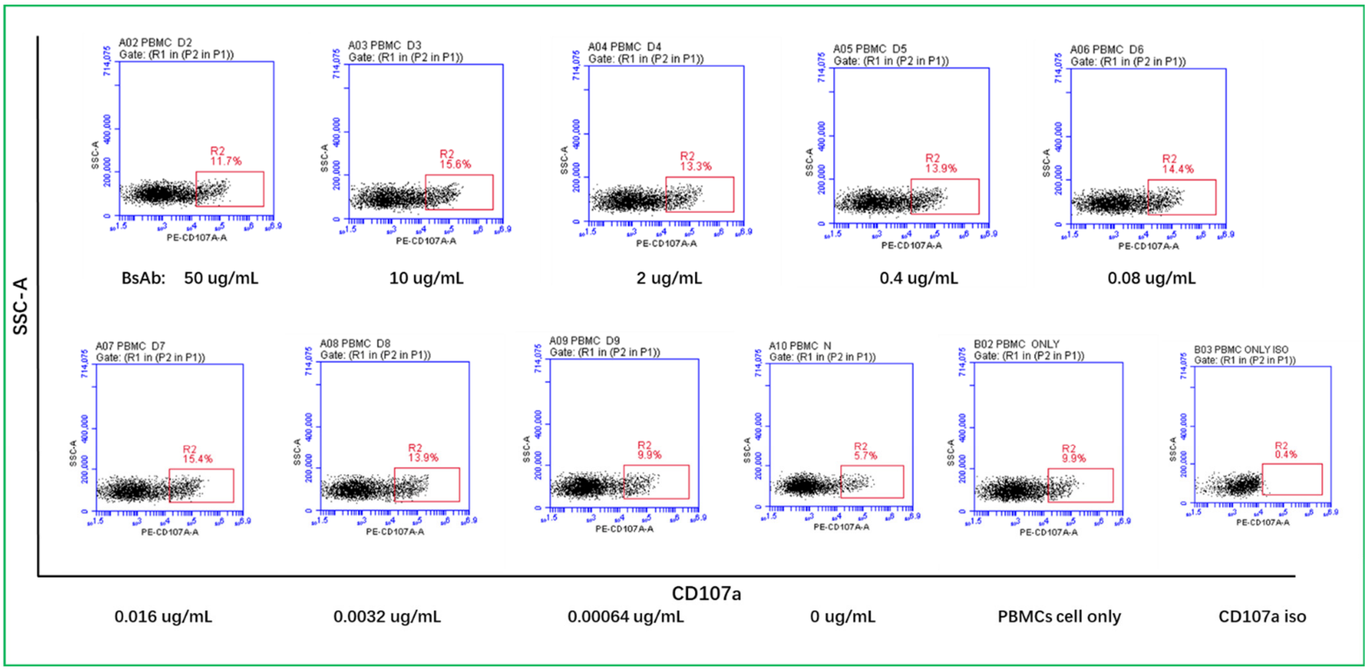

CD107a expression on CD3- CD56+ NK cells

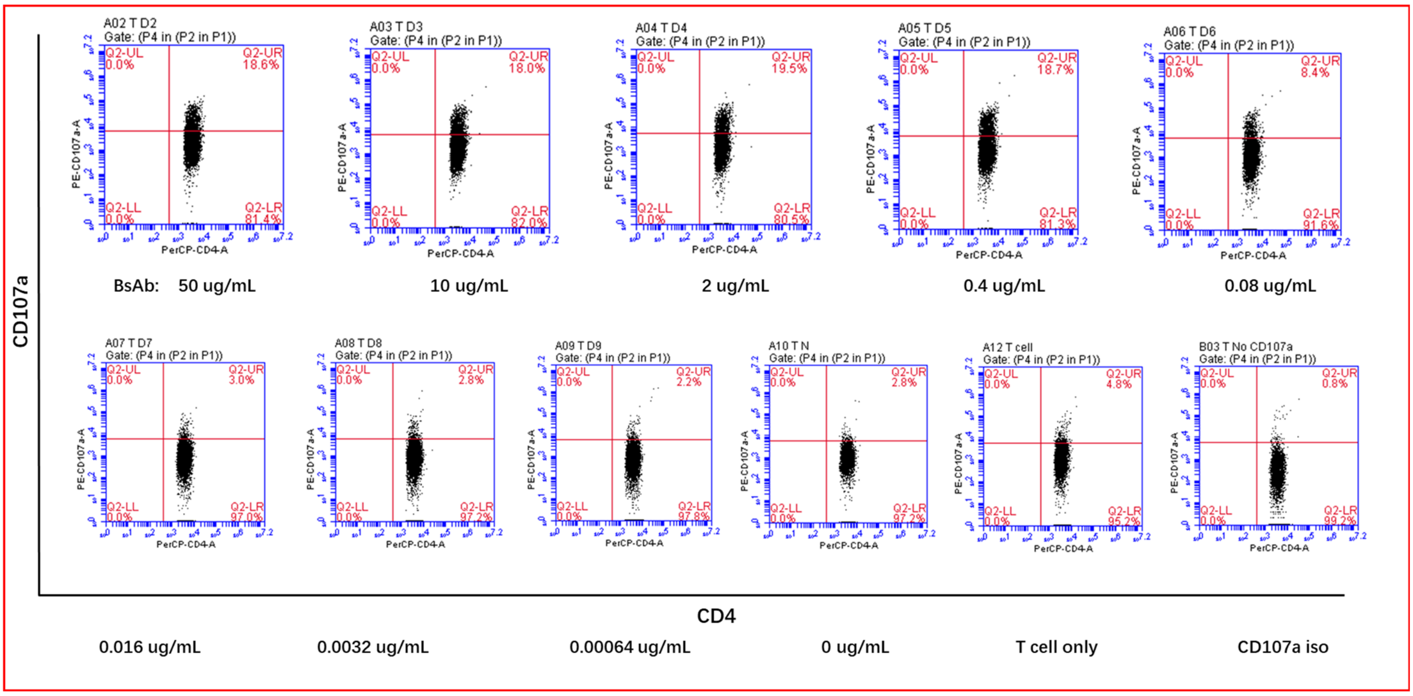

CD107a expression on CD4+ T cells

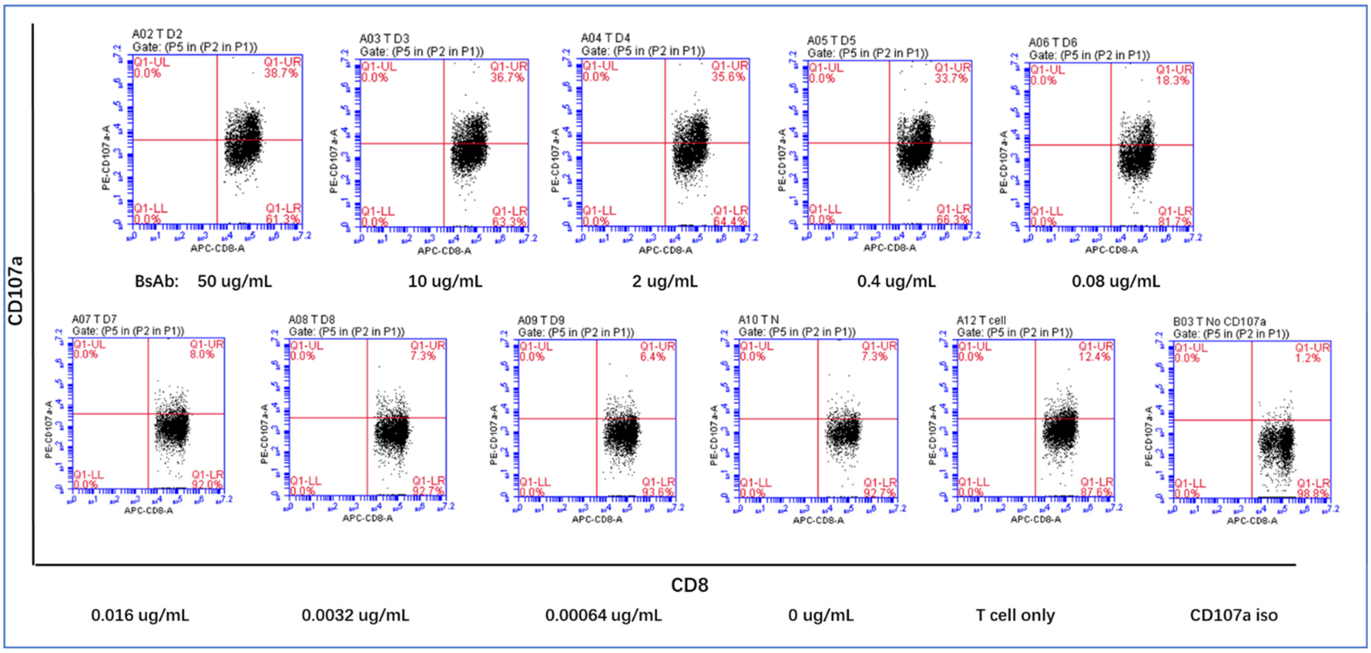

CD107a expression on CD8+ T cells
